# Machine learning based anoikis signature predicts personalized treatment strategy of breast cancer

**DOI:** 10.3389/fimmu.2024.1491508

**Published:** 2024-11-22

**Authors:** Xiao Guo, Jiaying Xing, Yuyan Cao, Wenchuang Yang, Xinlin Shi, Runhong Mu, Tao Wang

**Affiliations:** ^1^ School of Pharmacy, Beihua University, Jilin, China; ^2^ School of Basic Medical Sciences, Beihua University, Jilin, China; ^3^ Research Laboratory Center, Guizhou Provincial People’s Hospital, Guiyang, Guizhou, China

**Keywords:** breast cancer, anoikis, personalized treatment, PD-L1, methotrexate

## Abstract

**Background:**

Breast cancer remains a leading cause of mortality among women worldwide, emphasizing the urgent need for innovative prognostic tools to improve treatment strategies. Anoikis, a form of programmed cell death critical in preventing metastasis, plays a pivotal role in breast cancer progression.

**Methods:**

This study introduces the Artificial Intelligence-Derived Anoikis Signature (AIDAS), a novel machine learning-based prognostic tool that identifies key anoikis-related gene patterns in breast cancer. AIDAS was developed using multi-cohort transcriptomic data and validated through immunohistochemistry assays on clinical samples to ensure robustness and broad applicability.

**Results:**

AIDAS outperformed existing prognostic models in accurately predicting breast cancer outcomes, providing a reliable tool for personalized treatment. Patients with low AIDAS levels were found to be more responsive to immunotherapies, including PD-1/PD-L1 inhibitors, while high-AIDAS patients demonstrated greater susceptibility to specific chemotherapeutic agents, such as methotrexate.

**Conclusions:**

These findings highlight the critical role of anoikis in breast cancer prognosis and underscore AIDAS’s potential to guide individualized treatment strategies. By integrating machine learning with biological insights, AIDAS offers a promising approach for advancing personalized oncology. Its detailed understanding of the anoikis landscape paves the way for the development of targeted therapies, promising significant improvements in patient outcomes.

## Introduction

Breast cancer (BC) is the most common malignant tumor in women in the world, and its incidence rate has gradually increased in recent years (1). The diagnosis, treatment and prognosis of BC have a great impact on the health, lifestyle and work of individuals as well as their family life (2). With the continuous development of biomedical technology, the research on the prognosis of BC has also made much progress, and people’s awareness of personalized treatment is increasing (3). Multiple sets of data have been integrated to predict the prognosis of BC. For example, prediction models based on genomics, transcriptomics and proteomics data can be used to predict the survival rate and recurrence of BC patients (4). In recent years, artificial intelligence technology has also been widely used in predicting the prognosis of BC, and the prediction model based on machine learning can more accurately evaluate the prognosis by integrating a large number of clinical data and bioinformatics data (5).

Anoikis is a specialized form of programmed cell death triggered by the loss of cellular attachment to the extracellular matrix and neighboring cells, playing a pivotal role in tumor development and metastasis (6). While anoikis is crucial in tumor invasion and infiltration, there are limited studies systematically evaluating and predicting BC prognosis based on anoikis.

We conducted a comprehensive analysis to elucidate the importance of anoikis. Leveraging bulk and single-cell sequencing techniques, we evaluated anoikis activity across various cell types. Machine learning algorithms were employed to identify anoikis genes associated with BC prognosis, allowing us to construct predictive models. These models demonstrated the efficacy of anoikis in predicting BC patient outcomes, immune status, responsiveness to immune checkpoint inhibitors (ICIs) and chemotherapy, as well as in identifying potential therapeutic targets and drugs. Through rigorous evaluations, anoikis emerged as a promising tool for precise prognostication and treatment stratification in BC patients.

## Methods

### Data acquisition

We retrospectively collected data from 12 distinct breast cancer cohorts derived from The Cancer Genome Atlas (TCGA), Gene Expression Omnibus (GEO), and Metabric (7). These cohorts included samples with comprehensive survival information, enabling thorough analysis. Our study encompassed a total of 11,033 patients across the 12 cohorts for prognostic evaluation. The patient distribution was as follows: TCGA-BRCA (n = 1076), GSE202203 (n = 3206), GSE96058 (n = 3409), GSE20685 (n = 327), GSE58812 (n = 107), GSE21653 (n = 244), GSE7390 (n = 198), GSE11121 (n = 200), GSE86166 (n = 330), GSE88770 (n = 108), GE48391 (n = 81), and Metabric (n = 1747). Genes implicated in the anoikis process were obtained from the Molecular Signature Database on the GSEA website (8).

### Machine learning derived anoikis signature

To develop a breast cancer-specific anoikis signature, we employed the methodology established in our previous research (9). Our approach involved utilizing ten diverse computational Survival algorithms: Random Survival Forest (RSF), Least Absolute Shrinkage and Selection Operator (LASSO), Gradient Boosting Machine (GBM), Survival Support Vector Machine (Survival-SVM), Supervised Principal Component (SuperPC), Ridge Regression, Partial Least Squares Cox Regression (plsRcox), CoxBoost, Stepwise Cox regression, and Elastic Net (Enet). Among these, RSF, LASSO, CoxBoost, and Stepwise Cox were chosen for their ability to reduce dimensionality and identify relevant variables. These techniques were combined into 108 unique configurations to construct a predictive signature. By evaluating all cohorts, including TCGA and other datasets, we identified the most robust prognostic model through the calculation of the average Concordance index (C-index). This iterative process culminated in the creation of an anoikis-specific signature designed to predict outcomes in breast cancer.

### Genomic alteration analysis

To elucidate genetic disparities between the two AIDAS groups, we analyzed genetic mutation levels and Copy Number Alterations (CNA) using the TCGA-BRCA database. The Tumor Mutation Burden (TMB) for both high- and low-AIDAS breast cancer patients was derived from the raw mutation data. Utilizing the maftools landscape, we depicted the most frequently mutated genes (mutation rate > 5%). Patient-specific mutational signatures were identified using the deconstructSigs package (10), emphasizing four prominent mutational signatures (SBS3, SBS1, SB12, SBS11) that exhibited elevated mutation frequencies in the TCGA-BRCA dataset. We identified the five most common regions of amplification and deletion, with a specific focus on the four predominant genes in chromosomal regions 3q26.32 and 5q21.3.

### Single-cell data processing

We applied Seurat (v4.0) to process the single-cell data from GSE161529 (11). This involved filtering out genes with zero expression and retaining those with nonzero expression levels. The expression matrix was normalized using Seurat’s “SCTransform” function. Dimensionality reduction was performed using principal component analysis (PCA) and UMAP techniques. To identify distinct cellular groupings, we employed Seurat’s “FindNeighbors” and “FindClusters” functions. To ensure dataset integrity, the DoubletFinder package was used to eliminate potential doublets (12). Cells failing to meet quality standards, such as those with mitochondrial gene content exceeding 15% or fewer than 500 genes, were excluded. Following stringent quality control measures, 64,308 cells were retained for analysis. Cell types were determined by manual annotation based on established marker genes.

### Inference of regulons and their activity

We utilized the Single-Cell rEgulatory Network Inference (SCENIC) approach to construct gene regulatory networks (GRNs) from single-cell RNA sequencing data. SCENIC involves a three-step process: first, it identifies co-expression modules between transcription factors (TFs) and their potential target genes. Next, it identifies the direct target genes for each module, prioritizing those enriched with the motif of the associated TF, thereby defining a regulon comprising a TF and its direct targets. Finally, the regulatory activity score (RAS) is computed for each cell by evaluating the area under the recovery curve.

To address the conventional SCENIC protocol’s challenges with scalability for extensive datasets and its susceptibility to sequencing depth variations, we modified it to enhance both scalability and robustness. This involved partitioning the data into metacells before applying SCENIC to these gene expression profiles (13). This adjustment significantly improved data quality and reduced computational demands, representing a notable advancement in the application of SCENIC to single-cell RNA-seq data analysis.

### Regulon clustering

We employed a robust computational method to dissect the regulatory relationships between transcription factors (TFs) and their target genes, with a focus on TF clustering. The process began by filtering TF-target interaction data to isolate pairs exceeding a significance threshold (>1), prioritizing the most critical regulatory interactions. We then identified key regulatory TFs by assessing their influence on target gene regulation, highlighting them as central nodes in the regulatory network for detailed analysis.

To visualize the intricate network of TF-target interactions, we constructed a graph model. A force-directed algorithm was used to refine the spatial layout of the graph, intuitively representing the network’s structure and the interplay between TFs and their targets. For an enhanced understanding of the network’s architecture, the Leiden algorithm was applied for community detection. This revealed the modular organization of TFs based on their regulatory connections, assigning each TF to a specific cluster. This approach allowed for a detailed analysis of the regulatory landscape, providing insights into the functional organization of TFs within the network.

### Cell-cell communication analysis

Using the “CellChat” R package, we generated CellChat objects from the UMI count matrices for each group (14). The “CellChatDB.human” database was used as the reference for ligand-receptor interactions. Intercellular communication was interpreted using the default settings of the package. To compare interaction counts and intensities, we merged CellChat objects from each group with the “mergeCellChat” function. Differences in interaction numbers and intensities among specific cell types were visualized using the “netVisual_diffInteraction” function. Changes in signaling pathways were identified using the “rankNet” function, and the distribution of signaling gene expression among groups was displayed with the “netVisual_bubble” and “netVisual_aggregate” functions.

Additionally, we employed the NicheNet package to analyze intercellular communication from the perspective of ligand activity and the expression patterns of specific downstream targets regulated by these key ligands (15). This method provided a detailed understanding of the signaling processes underlying cell-cell interactions, using ligand-target relationships to infer communication pathways within the cellular microenvironment.

### Evaluation of TME disparities and immunotherapy response

To comprehensively and accurately assess immune cell infiltration levels, we analyzed adverse infiltrated immune cells using multiple algorithms, including MCPcounter, EPIC, xCell, CIBERSORT, quanTIseq, and TIMER, among patients stratified by the AIDAS (16–22). Additionally, to precisely depict the immune landscape and architecture within the tumor microenvironment (TME), we evaluated the ESTIMATE and TIDE indices. These metrics provide critical insights into the potential for immunotherapy and offer prognostic implications for breast cancer patients.

Moreover, we quantified immune checkpoints, which serve as indicators of the immune state and offer preliminary predictions of patient responsiveness to ICI therapy. This comprehensive evaluation of the immune profile within the TME is crucial for advancing personalized medicine and refining treatment strategies for breast cancer patients.

### Determination of therapeutic targets and drugs for high AIDAS patients

We identified therapeutic targets and drugs for high-AIDAS patients from the Drug Repurposing Hub and dropped out duplicate compounds, resulting in a refined list of 6,125 compounds. We established the selection of therapeutic targets associated with breast cancer outcomes through Spearman correlation analysis. Specifically, we assessed the relationship between the AIDAS and gene expression levels, selecting genes with a correlation coefficient greater than 0.3 and a P-value less than 0.05. Additionally, genes with a correlation coefficient below -0.3 and a P-value below 0.05 were identified as linked to poor prognosis. The significance of these genes was further evaluated by examining the relationship between CERES scores from the Cancer Cell Line Encyclopedia (CCLE) and model value (23).

To enhance predictions regarding drug responsiveness, we utilized data from the Cancer Therapeutics Response Portal (CTRP) and the PRISM project, both of which offer extensive drug screening and molecular data across diverse cancer cell lines. Differential expression analysis was conducted between bulk samples and cell lines. Subsequently, we employed the pRRophetic package to implement a ridge regression model for predicting drug response. This model, trained using expression data and drug response metrics from solid Cancer Cell Lines (CCLs), demonstrated excellent predictive accuracy, validated through 10-fold cross-validation (24).

Furthermore, to identify the most promising therapeutic drugs for breast cancer, we performed a Connectivity Map (CMap) analysis. This entailed comparing gene expression profiles across different risk subgroups and submitting the top 300 genes (150 up-regulated and 150 down-regulated) to the CMap website. A negative CMap score indicated a higher therapeutic potential against breast cancer, suggesting an inverse relationship between the CMap score and a compound’s effectiveness as a potential treatment.

### Patient stratification

To evaluate gene expression in breast cancer specimens, RNA extraction was conducted using TRIzol reagent (Invitrogen, Carlsbad, CA, USA). This was followed by cDNA synthesis and quantitative reverse transcription PCR (qRT-PCR) using GoScript reverse transcriptase and Master Mix (Promega), adhering to the manufacturer’s instructions. Data acquisition was performed with the CFX96 Touch Real-Time PCR Detection System (BioRad, Hercules, CA, USA). Gene expression levels were quantified using the 2^-ΔΔCq^ method, with GAPDH serving as the normalization control. Patients were subsequently categorized based on their gene expression profiles using a predefined formula derived from the AIDAS. This stratification was crucial in identifying patients with distinct risk profiles, thus facilitating tailored therapeutic interventions.

### Immunohistochemistry experiment

Tissue samples were collected from 30 breast cancer patients undergoing surgery at Guizhou Provincial People’s Hospital. These samples were subjected to Hematoxylin and Eosin (H&E) staining following established protocols (25, 26), with diagnoses independently confirmed by two pathologists.

For immunohistochemistry (IHC) analysis, paraffin-embedded samples were processed according to procedures outlined in previous studies. Protein expression levels were evaluated independently by two pathologists, adhering to standardized protocols and scoring systems consistent with methodologies from prior research (26).

## Results

### Construction of an anoikis model using artificial intelligence

**Figure 1:**
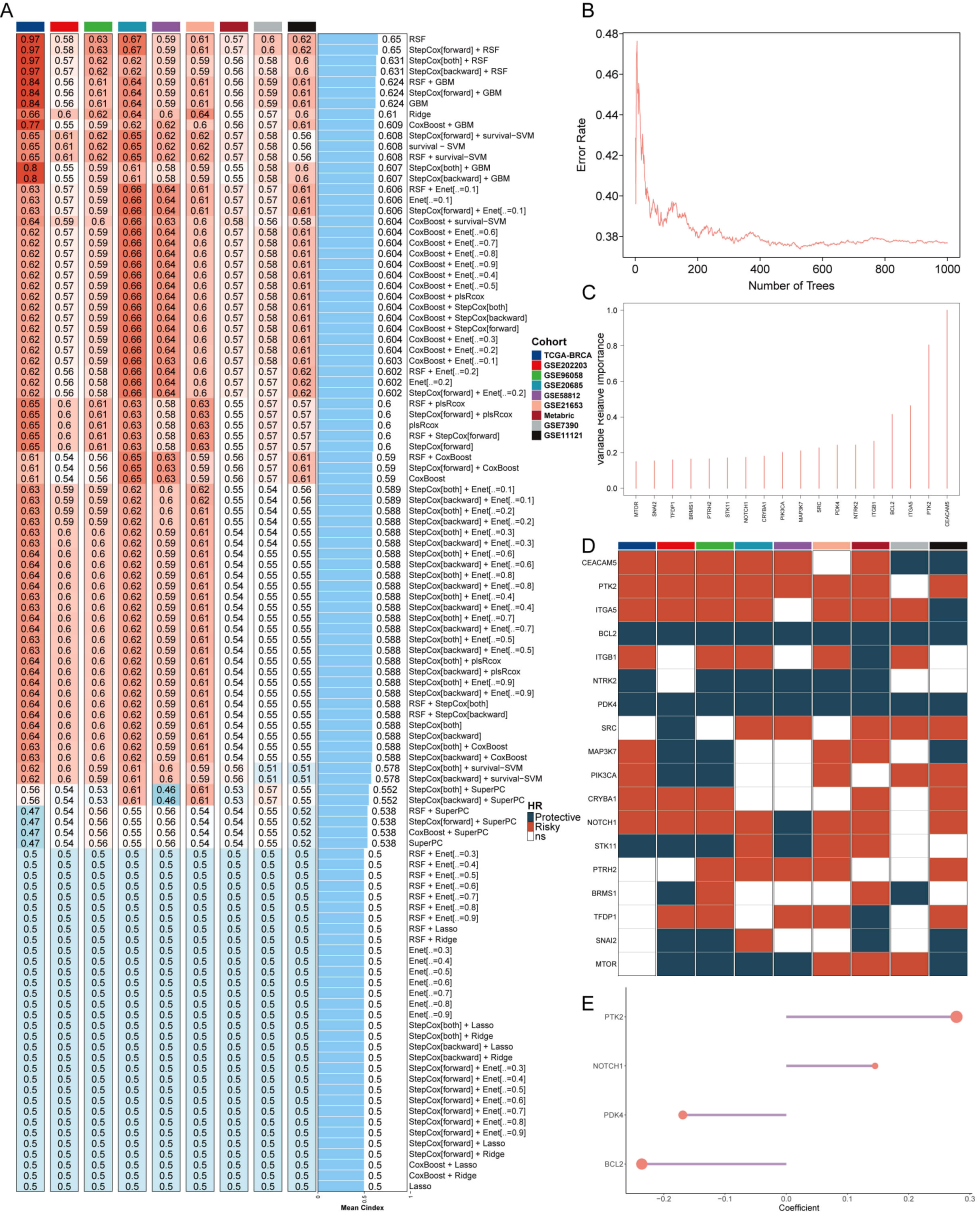
Construction of an anoikis model using artificial intelligence. **(A)** C-indexes of the 108 machine learning algorithm combinations in the nine cohorts. **(B)** Error rate of RSF after 1000 tests. **(C)** Key anoikis genes selected by RSF. **(D)** Prognostic value of key genes in nine BC cohorts. **(E)** Final selection of 4 anoikis genes based on an exhaustive search, with patient risk scores calculated according to the expression levels of these genes and their regression coefficients.

The comprehensive evaluation of the anoikis model was conducted using a combination of 108 machine learning algorithms with ten-fold cross-validation (**Figure 1A**). The performance of the models was assessed by calculating the average C-index across various cohorts, with the Random Survival Forest (RSF) algorithm demonstrating the highest average C-index (0.632). The key anoikis genes were identified based on the point with the lowest error rate of RSF in 1000 tests (**Figures 1B, C**). These genes underwent univariate Cox regression analysis to calculate the hazard ratio (HR) across nine enrolled cohorts (**Figure 1D**). Finally, four genes (PTK2, coef = 0.278; NOTCH1, coef = 0.145; PKD4, coef = -0.169; BCL2, coef = -0.236) were selected to construct an artificial intelligence-derived anoikis signature (AIDAS) (**Figure 1E**). The evaluation of AIDAS across the nine cohorts revealed that the binary classification model effectively classified patients into high and low-AIDAS groups (**Supplementary Figure S1**).

### Assessment of AIDAS with 83 published models

We further conducted both univariate and multivariate Cox analysis to assess the independence of AIDAS and other clinical indices (**Supplementary Figure S2A**). Three significant indices, namely AIDAS, stage, and age, were chosen to develop a nomogram aimed at predicting patients’ survival rates in clinical practice (**Supplementary Figure S2B**). The overall survival (OS) of breast cancer patients with different conditions was predicted, and the OS curve demonstrated a good fit with the standard curve, indicating the model’s accuracy (**Supplementary Figures S2C, D**). Through comparisons with other factors, it was observed that AIDAS could provide more accurate predictions of patients’ conditions (**Supplementary Figure S2F**).

**Figure 2:**
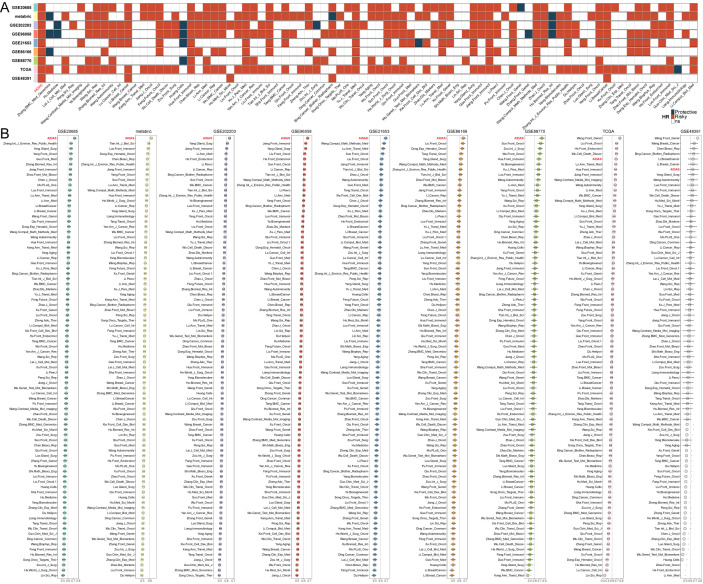
Assessment of AIDAS with 83 published models. **(A)** The stability of AIDAS was compared with 83 published models. **(B)** C-index values of AIDAS and 83 published models in 9 different datasets.

The stability of the predictive model of the AIDAS was evaluated by collecting and assessing 83 published signatures in BC across 9 independent cohorts. It was demonstrated that only the AIDAS exhibited consistent statistical significance across all cohorts (**Figure 2A**). The predictive power of the AIDAS was compared with the 83 models across the 9 cohorts using the C-index (**Figure 2B**). The AIDAS showed significantly better accuracy than the others in almost all cohorts, ranking first in seven cohorts, fifth in one cohort, and seventh in one cohort, thereby revealing the stability of our model (**Figure 2B**).

### Multi-omics analysis of genomic alterations based on AIDAS

**Figure 3:**
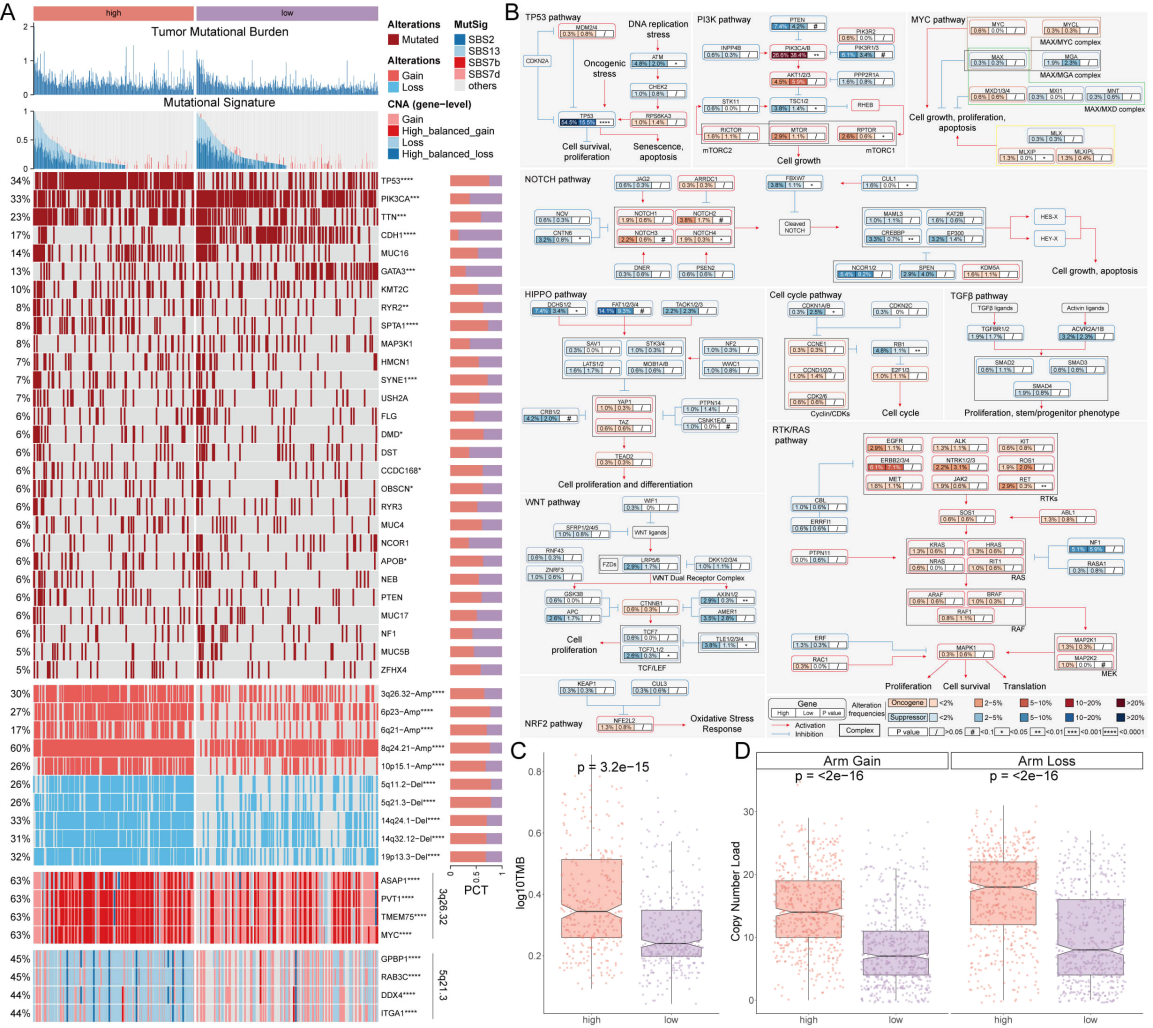
Multi-omics analysis of genomic alterations based on AIDAS. **(A)** Overview of genomic variations based on AIDAS. **(B)** Mutation atlas of 10 oncogenic pathways. **(C)** Difference of TMB values. **(D)** Comparison of copy number load between the two AIDAS groups. *P < 0.05; **P < 0.01; ***P < 0.001; ****P < 0.0001.

Gene variations between the AIDAS groups were analyzed using multi-omics integration analysis. We observed a significant increase in TMB in high-AIDAS patients, accompanied by multigene mutation characteristics (**Figures 3A, C**). When considering 10 oncogenic signaling pathways together, classic tumor suppressor genes like TP53, RB1, and AXIN1/2 were found to mutate more frequently in the high-AIDAS group, while oncogenic genes such as RET, PIK3CA/B, and RPTOR mutated less (**Figures 3A, B**). Further analysis of CNV between these subgroups revealed that amplifications and deletions at the level of chromosome arms were more pronounced in the high-AIDAS group, including amplifications of 3q26.32, 6p23, 6q21, 8q24.21, and 10p15.1, as well as deletions of 5q11.2, 5q21.3, 14q24.1, 14q32.12, and 19p13.3 (**Figures 3A, D**). These results suggest that the poor prognosis for high-AIDAS patients may be related to significant increases in the amplification of 3q26.32 and multiple oncogenes genes (ASAP1, PVT1, TMEM75, and MYC), as well as deletions of multiple tumor suppressor genes of 5q21.3 (GPBP1, RAB3C, DDX4, and ITGA1) (**Figure 3A**).

### Deciphering the AIDAS at the single-cell level

**Figure 4:**
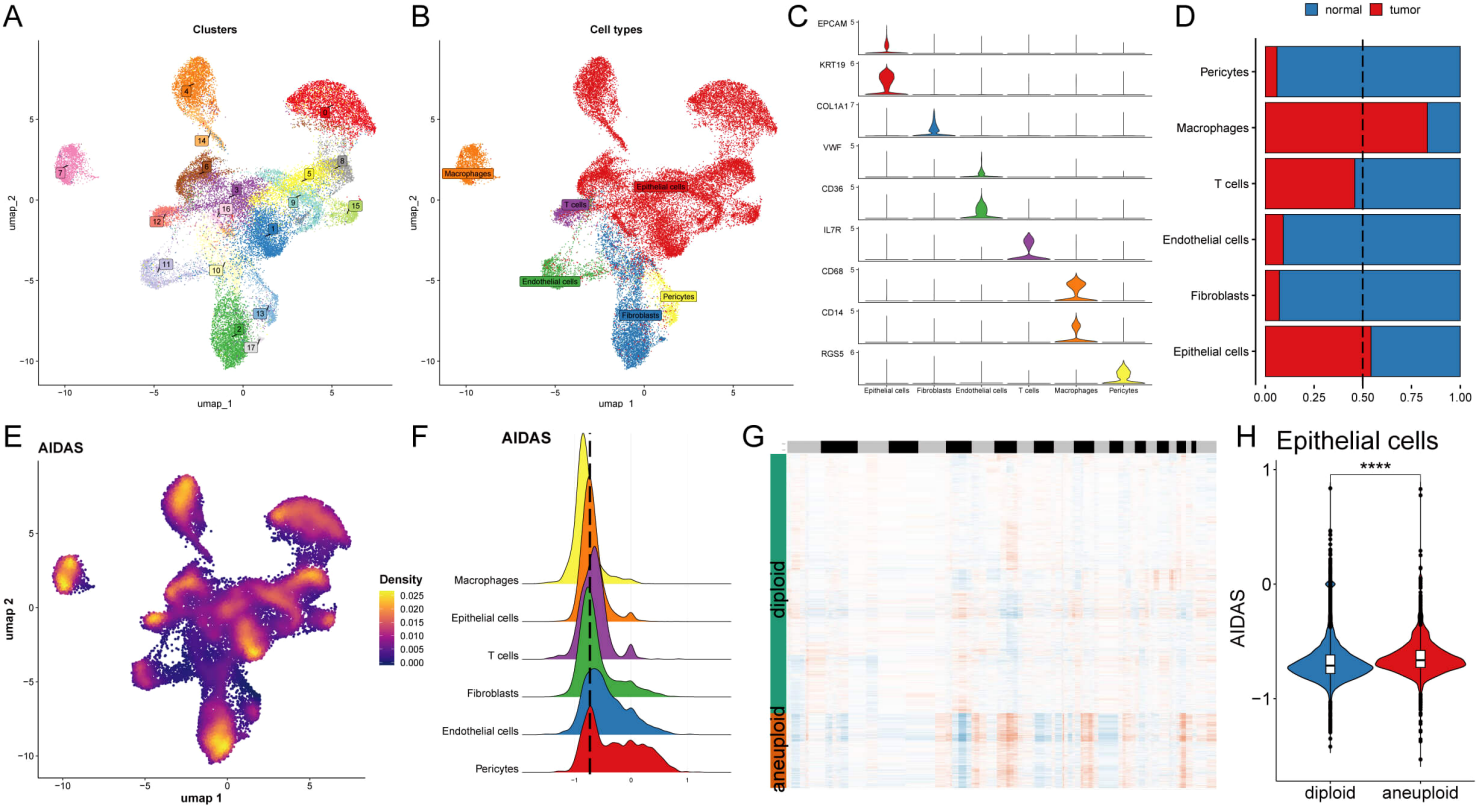
Deciphering the AIDAS at the single-cell level. **(A)** Distribution of 20 cell clusters. **(B)** Seven cell types identified by the established marker genes. **(C)** Representative markers of each cell type. **(D)** Proportion of seven types of cells between tumor patients and normal tissues; **(E)** Distribution of AIDAS value. **(F)** AIDAS value across cell types. **(G)** CNV evaluation using copyKat algorithm. **(H)** Comparison of the AIDAS value between diploid and aneuploid cells within the epithelial cells. ^****^P<0.0001.

The expression characteristics of different immune infiltrating cells were revealed at the single-cell level. The distribution of cells from 8 BC patients was analyzed, and the distribution of tumor and normal tissues (**Supplementary Figures S3A, B**), 17 cell clusters were identified and divided into 6 cell types (**Figures 4A, B**). The number of cells in these 6 types was statistically analyzed, and then their proportion in the bodies of these 8 tumor patients was calculated (**Supplementary Figures S3C, D**). The representative markers in these 6 types of cells, as well as their actual distribution in the cells (**Figure 4C**; **Supplementary Figure S3E**) were observed. The results showed that epithelial cells and macrophages accounted for a larger proportion of the tumor tissue, while fibroblasts, T cells, Pericytes, and endothelial cells accounted for a larger proportion in the normal tissue (**Figure 4D**).

Next, the AIDAS was incorporated into the single-cell distribution map (**Figure 4E**). All cells were divided into low- and high-AIDAS groups based on their peak of epithelial cells (**Figure 4F**), and then differential gene expression analysis and functional clustering were performed to elucidate potential functional pathways (**Supplementary Figures S3F, G**). Subsequently, copyKat analysis was performed to observe the CNV for distinguishing the normal cells and tumor cells (**Figure 4G**). We observed a higher AIDAS score in tumor-aneuploid than in tumor-diploid, implying the significance of AIDAS in breast cancer progression (**Figure 4H**).

### Specific regulons for AIDAS and cell recognition

**Figure 5:**
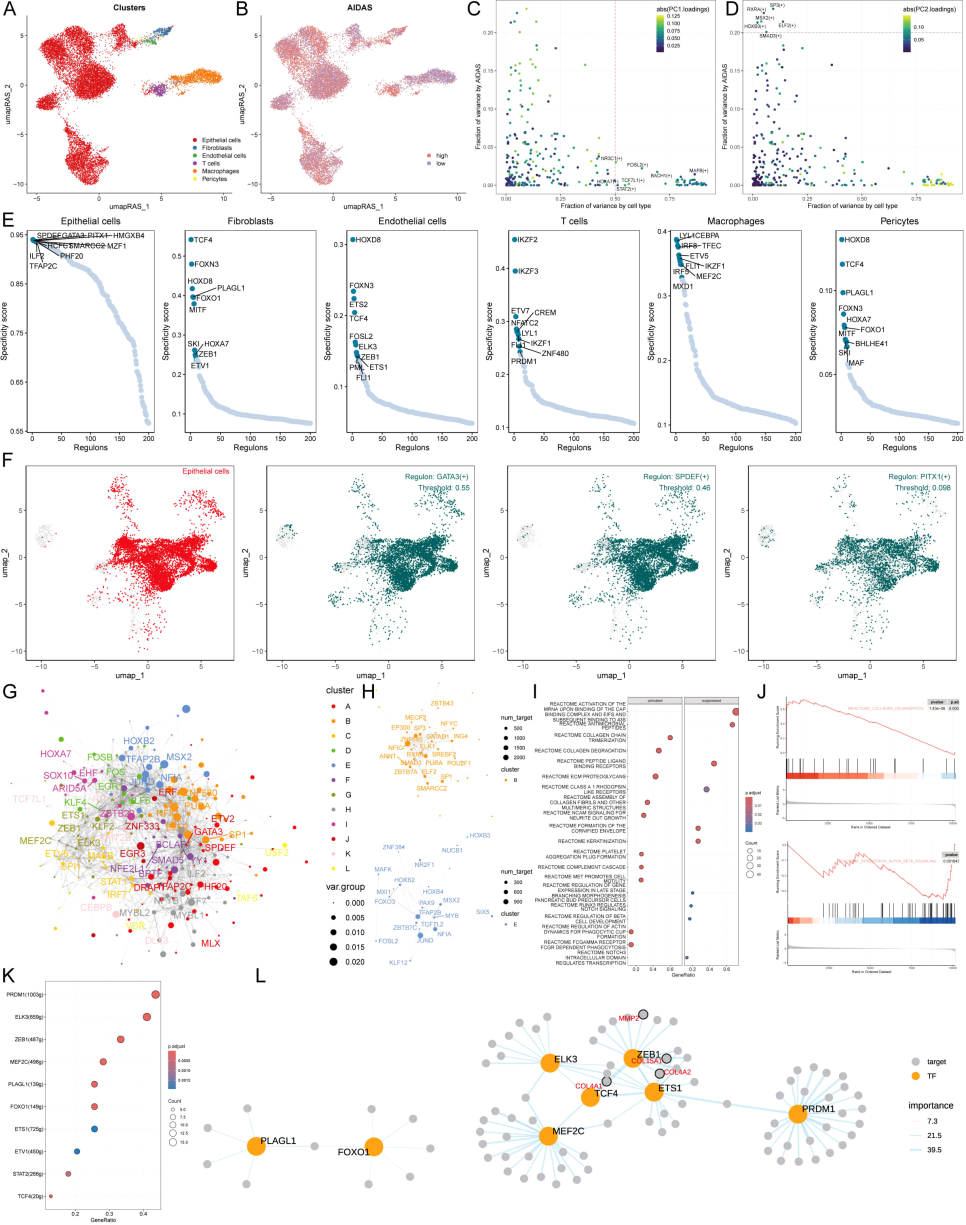
Specific regulons for AIDAS and cell recognition. **(A)** Distribution of cell types based on RAS. **(B)** Distribution of AIDAS value based on RAS. **(C)** Variance analysis highlights the impact of cell types and AIDAS on transcription factor activity, using color mapping to PC1 to emphasize the primary variance influenced by these factors. **(D)** Variance analysis with color mapped to PC2. **(E)** Scores of specific transcription factors in different types of cells. **(F)** Specific distribution of the most relevant specific regulators in epithelial cells. **(G)** Network of each transcription factor based on Leiden algorithm. **(H)** Specific transcription factor groups with higher scores in AIDAS. **(I)** GSEA identifies pathway variations linked to AIDAS in epithelial cells. **(K)** Transcription factors that could contribute to the collagen degradation. **(L)** Network of the regulatory relationship related to collagen degradation.

To comprehensively construct a GRNs of AIDAS, a SCENIC pipeline was applied to analyze single-cell RNA seq data with cis-regulatory sequence information (**Figures 5A, B**). PCA and variance analyses were performed on different cell types and AIDAS. PCA1 explained the specific transcription factors of different cell types, while PCA2 was associated with the regulons of AIDAS (**Figures 5C, D**). The key transcription factors for cell recognition were identified, and the regulon specificity score (RSS) of these specific transcription factors in different types of cells was evaluated (**Figure 5E**). The regulatory factors with higher RSS scores were selected from these six types of cells, and GATA3, SPDEF, and PITX1 were identified as the most relevant specific regulators to epithelial cells (**Figure 5F**). Similarly, the most relevant specific regulators to the other five types of cells were analyzed (**Supplementary Figure S4A**).

Understanding that TFs often collaborate to modulate gene expression, we systematically explored the combinatory patterns of these regulatory elements. Based on the Leiden algorithm, the similarity of RAS scores for each TF was compared, and the cluster analysis of TFs was conducted to find 12 clusters of transcription factors, of which the contribution rate of transcription factor sets B and E to the development of AIDAS was greater than that of the other 11 clusters, so only transcription factors B and E were displayed (**Figures 5G, H**, **Supplementary Figure S4B**). We next focused on the exact TFs that drive epithelial cells’ transcriptomic changes by AIDAS. Multiple pathways were identified by GSEA analysis. For example, collagen degradation was activated in epithelial cells in the high-AIDAS cells, while interference alpha beta signaling was inhibited (**Figures 5I, J**). Transcription factors contributing to these pathways were identified by further analysis (**Figure 5K**). The network diagrams of regulatory relationships among transcription factors were shown (**Figure 5L**).

### Intercellular communications for AIDAS

**Figure 6:**
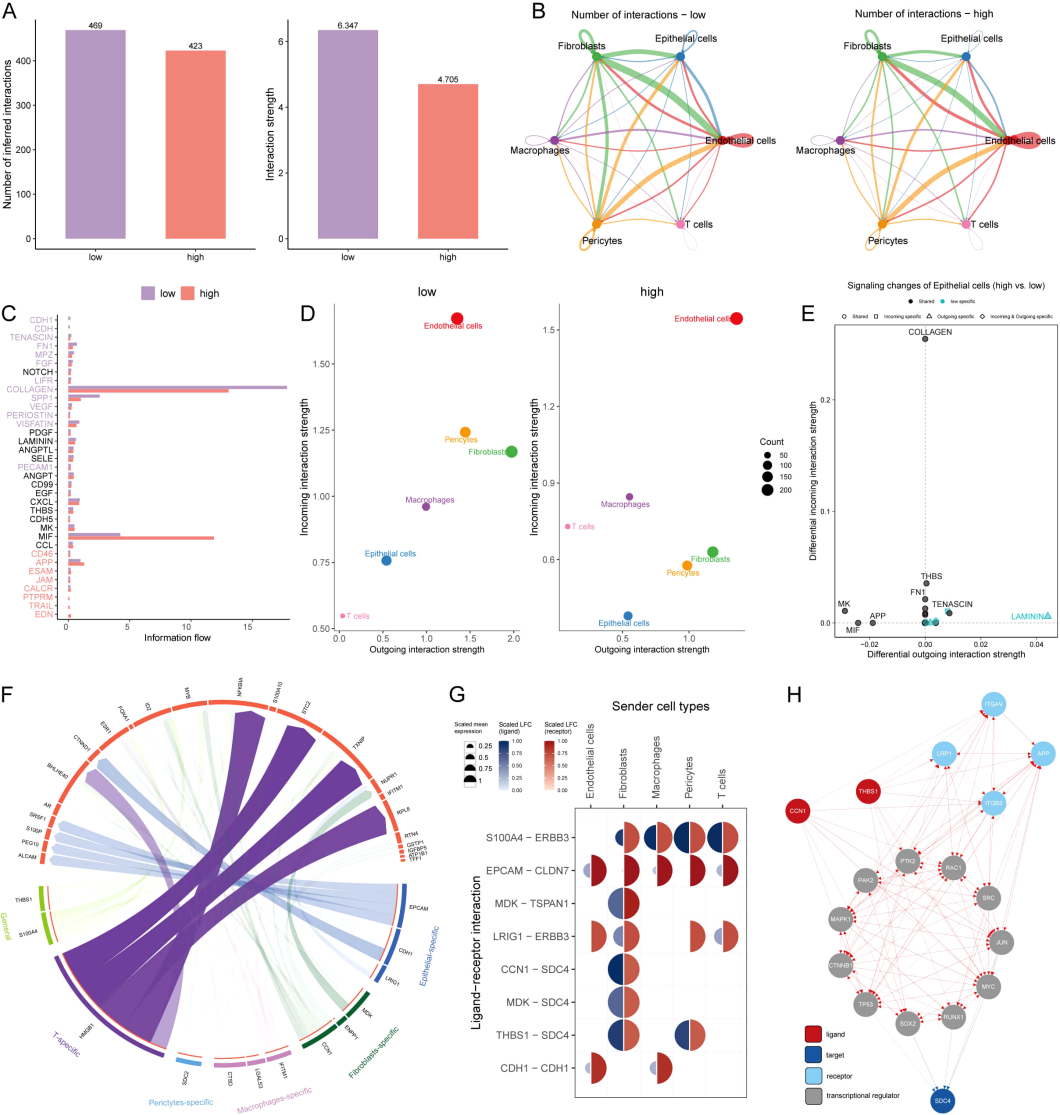
Intercellular communications for AIDAS. **(A)** Comparison of the number and intensity of cell interactions between two AIDAS groups. **(B)** Detail of cell communications among each cell type. **(C)** Differences of signaling pathways involved in the intercellular communication. **(D)** Intensity of incoming and outgoing interactions among different cells. **(E)** Specificity of incoming and outgoing signals of different signaling pathways. **(F)** Specific regulatory of ligands and receptors in cells. **(G)** Expression levels of ligands and receptors in different cells. **(H)** Route diagram of reaching target receptor SDC4 of CCN1 and THBS1 ligands through other receptors or transcription factors.

Intercellular communication among six cell types was evaluated by CellChat analysis. We observed that the number and intensity of cell-cell interactions were stronger in the low-AIDAS cells, and the intercellular communication between epithelial cells and endothelial cells was elevated (**Figures 6A, B**). Some signaling pathways involved in intercellular communication were analyzed, and the results showed that most of them had stronger intercellular communications in the low-AIDAS cells (**Figure 6C**). By comparing changes in outgoing and incoming signals among different cells, it was found that incoming interactions of epithelial cells were stronger in the low-AIDAS cells, indicating that incoming interactions of epithelial cells in the low-AIDAS group may be enhanced after they receive signals from other cells (**Figure 6D**).

Potential ligands of epithelial cells in the different groups were speculated using nichenetr analysis. We inferred potential ligands that may regulate epithelial cells from other cells based on the AIDAS group. The potential ligand-receptor pairs were further evaluated (**Figure 6F**). A high degree of interaction between THBS1-SDC4 and CNN1-SDC4 was observed, indicating that fibroblasts are the main sending cells affecting changes in the epithelial cell pathway (**Figure 6G**). THBS1 ligand and CNN1 ligand could reach the SDC4 through other receptors or transcription factors, in which high mutation rates of transcription factors such as TP53, MYC, and RAC1 in high-AIDAS (**Figure 6H**).

### Personalized immunotherapy for low-AIDAS patients

**Figure 7:**
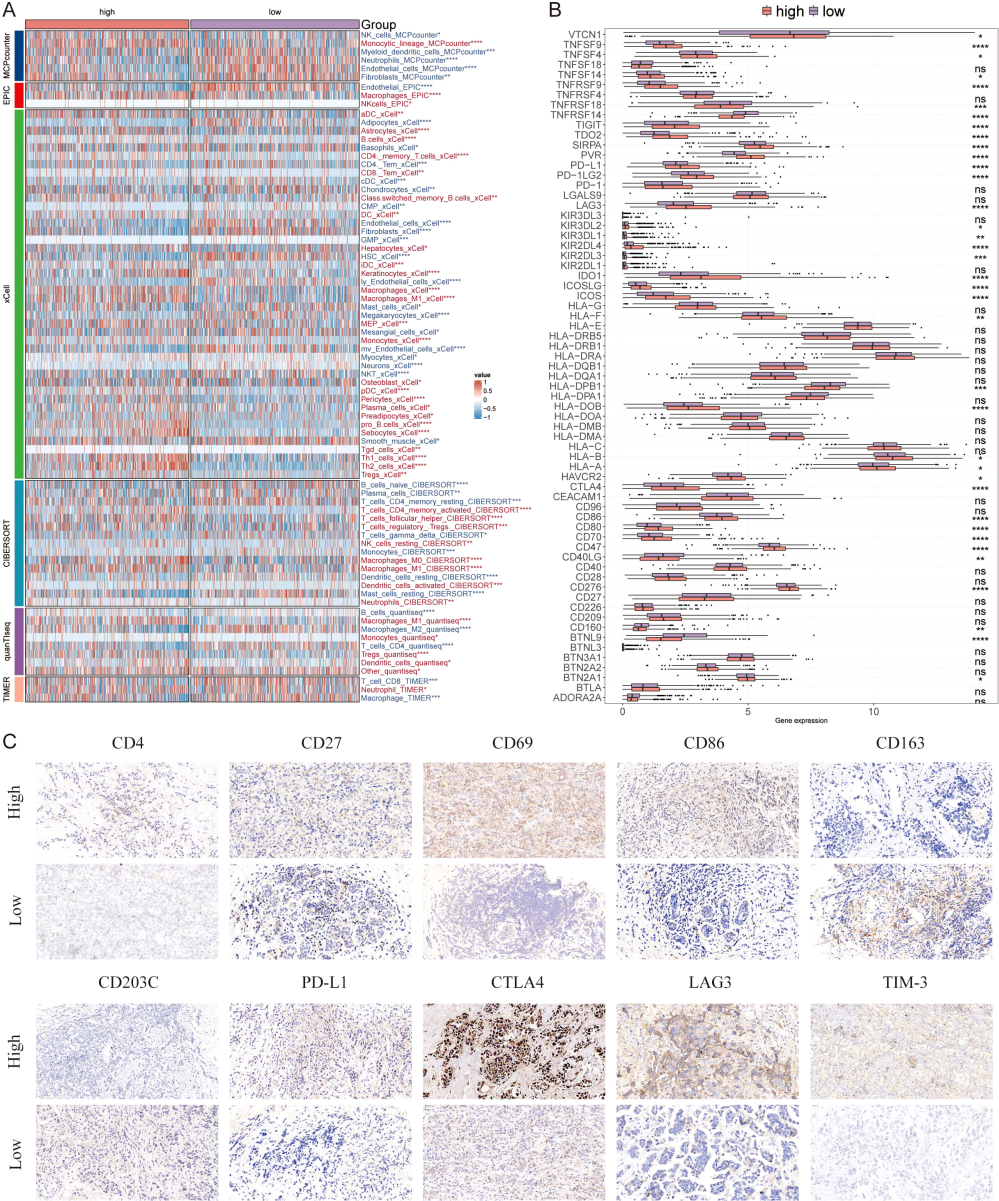
Differential expression and immunohistochemical analysis of immune markers in tumor microenvironments between AIDAS subgroups. **(A)** Heatmap provides a comparative view of immune cell infiltration in tumor samples with low and high AIDAS, utilizing various computational algorithms for quantification. Each row represents a different type of immune cell, with the color intensity reflecting the level of infiltration. **(B)** Box plots illustrate the distribution of gene expression levels for ICIs across low *vs*. high AIDAS conditions, with statistical significance denoted by ns for not significant; *P < 0.05; **P < 0.01; ***P < 0.001; ****P < 0.0001. **(C)** Representative immunohistochemistry images showcase the staining intensity of various immune markers between high and low expression conditions, visually depicting the differential expression of these markers in correlation with AIDAS levels.

Immune microenvironment is involved in breast cancer progression, six algorithms were applied to evaluate the immune infiltration of different AIDAS patients. A higher proportion of memory T cells, Tregs, M1 macrophages, and CD8^+^ T cells were observed in the high-AIDAS patients (**Figure 7A**), and some ICIs were also overexpressed, such as PD-L1, CTLA4, and LAG3 (**Figure 7B**). IHC was performed to support the above results using the representative cell markers and clinical ICIs (**Figure 7C**).

**Figure 8:**
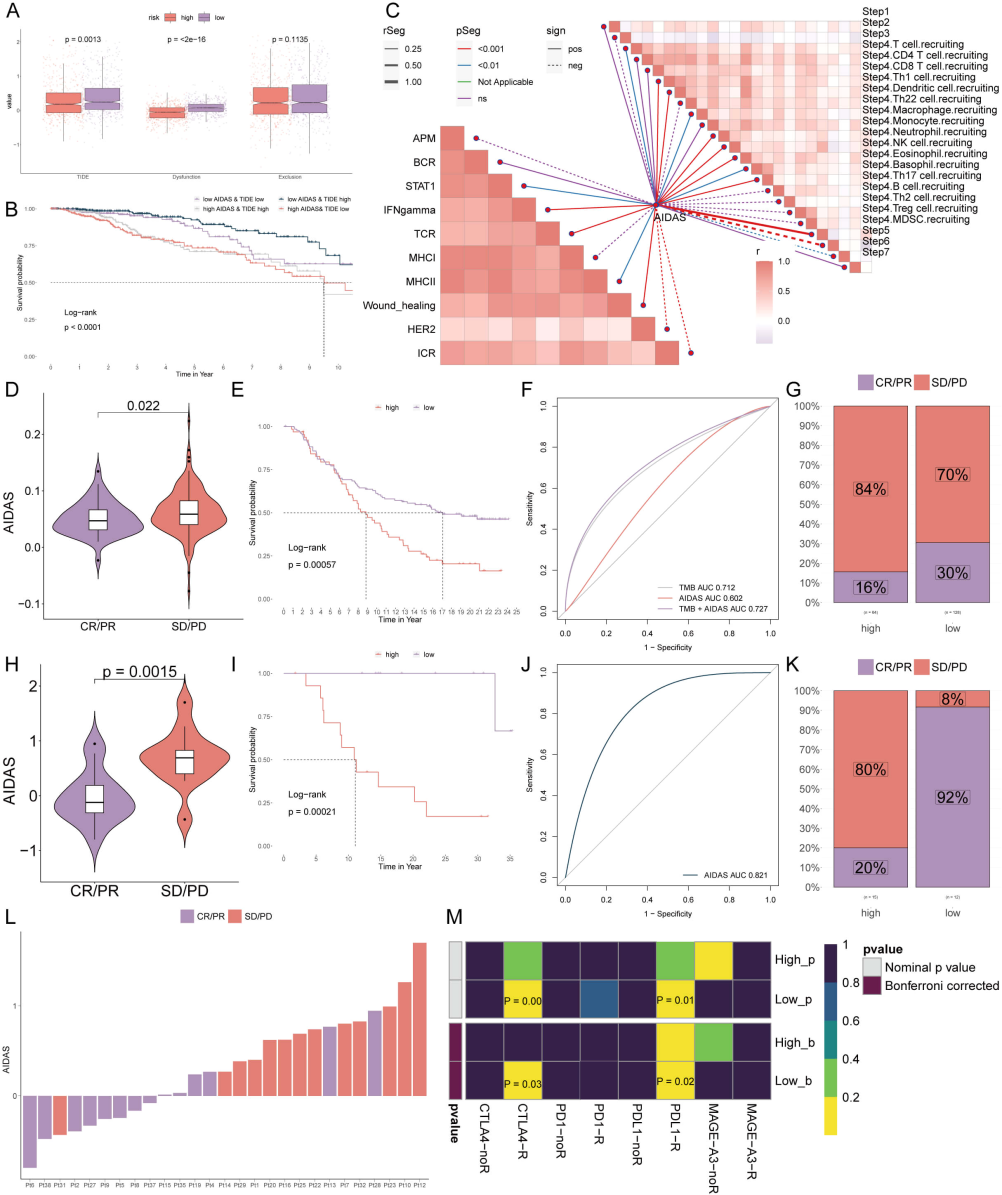
Personalized immunotherapy for low-AIDAS patients. **(A)** Differences in TIDE, Dysfunction, and Exclusion between patients in the low- and high-AIDAS groups. **(B)** Comparison of the survival probability of four combinations. **(C)** Correlation analysis of AIDAS with tumor immune cycle and ten immune pathways. **(D)** Correlation analysis of AIDAS value with anti-PD-L1 response. **(E)** KM survival curves of AIDAS after anti-PD-L1 treatment. **(F)** Accuracy of AIDAS and TMB in anti-PD-L1 treatment. **(G)** Proportion of CR/PR and SD/PD of anti-PD-L1 in ADIAS subgroups. **(H)** Correlation analysis of AIDAS value with anti-PD-1 response. **(I)** KM survival curves of AIDAS after anti-PD-1 treatment. **(J)** Accuracy of AIDAS and TMB in anti-PD-1 treatment. **(K)** Proportion of CR/PR and SD/PD of anti-PD-1 in ADIAS subgroups. **(L)** Distribution of ADIAS score of different patients after anti-PD-1 treatment. **(M)** Heatmap demonstrating the predictive power of ADIAS for responsiveness to different ICIs treatment.

Further analysis revealed that TIDE and Dysfunction values in the low-AIDAS group were higher than those in the high-AIDAS group, and there was no significant difference in the Exclusion value between the two groups (**Figure 8A**). There was a longer survival time in patients with a low-AIDAS and high-TIDE than in other combinations (**Figure 8B**). The correlation of AIDAS with the immune cycle and signaling showed that the anti-tumor immune activity of low-AIDAS patients was higher than that of high-AIDAS patients (**Figure 8C**).

ICIs have emerged as a transformative approach in cancer immunotherapy over the past several decades, yet their effectiveness in solid tumors, including breast cancer, remains limited. We sought to explore the predictive capability of AIDAS levels regarding the efficacy of immune checkpoint blockade therapies in the IMvigor210 (anti-PD-L1) and GSE78220 (anti-PD-1) cohorts.

Patients from low-AIDAS presented remarkable clinical benefits and better survival rates than the high-AIDAS in anti-PD-L1 response (**Figures 8D–G**). Prior benefits for low-AIDAS patients were also observed in anti-PD1 response (**Figures 8H–L**). Utilizing SubMap algorithms, we confirmed the response to immunotherapy, which was significantly more likely to benefit from treatments with anti-PD-L1 and CTLA4 treatments (**Figure 8M**). Based on the above research results, patients with the low-AIDAS can achieve better results in the treatment with ICIs.

### Identification of therapeutic drugs for high-AIDAS patients

**Figure 9:**
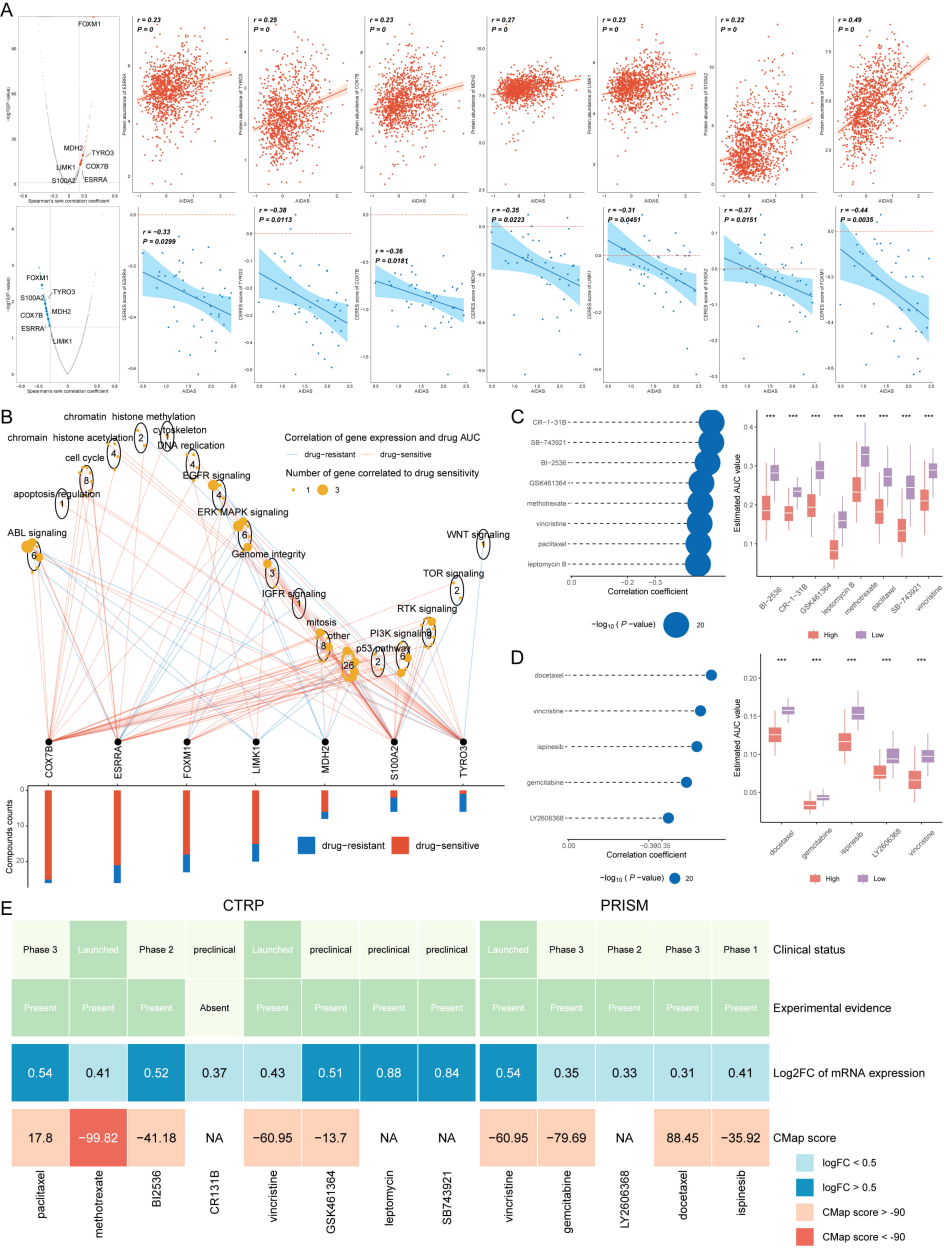
Identification of therapeutic drugs for high-AIDAS patients. **(A)** Spearman correlation of 7 potential therapeutic targets, where red and blue represent positive and negative correlations, respectively. **(B)** Network analysis highlighting the connections between the 7 therapeutic targets and their related drug action pathways. **(C)** AUC values of identified compounds from CTRP database. **(D)** AUC values of identified compounds from PRISM database. **(E)** Analysis from multiple perspectives based on the clinical status, experimental evidence, mRNA expression, and CMap score of 13 compounds. ***P < 0.001.

Chemotherapy is the standard treatment for anti-cancer, and data from multiple datasets have been used to develop potential drugs for BC patients with high-AIDAS. Seven therapeutic targets were identified using Spearman correlation analysis, and the results showed that high-AIDAS patients were positively correlated with the abundance of seven genes (MDH2, LIMK1, S100A2, TYRO3, COX7B, and ESRRA), and significantly negatively correlated with their CERES scores, suggesting that these seven genes can serve as a potential therapeutic target (**Figure 9A**). Potential drug targets were further analyzed based on drug sensitivity ratios, and it was revealed that these 7 genes had a high sensitivity to the drugs, so they were considered the key therapeutic targets for high-AIDAS patients (**Figure 9B**). Thirteen compounds were screened out from CTPR (CR-1-31B, SB-743921, BI2536, GSK461364, methotrexate, vincristine, paclitaxel, and leptomycin B) and PRISM datasets (docetaxel, vincristine, ispinesib, gemcitabine, and LY2606368), for evaluating candidate therapeutic drugs. The AUC values of the different compounds in the two groups were compared, and the results showed that lower AUC values were identified in high-AIDAS patients, indicating that these compounds may be suitable for the drug treatment of high-AIDAS patients (**Figures 9C, D**). The promising therapeutic agents were identified by CMap analysis, in which methotrexate, with a CMAP value of -99.82, was ultimately identified as the best potential therapeutic drug for high-AIDAS patients (**Figure 9E**).

## Discussion

Considering the unique clinical characteristics of BC patients, it is necessary to customize specialized prognostic plans for these patients, and it is crucial to develop an accurate prognostic model. Anoikis is a specific form of programmed apoptosis caused by the disruption of cell-cell or cell-extracellular matrix attachment, and eliminating displaced or displaced cells can help maintain the dynamic balance of tissues (27), Anoikis is a term that describes the process of apoptosis that triggered by the detachment of cells from the extracellular matrix (28). It has been confirmed that anoikis is the first line of defense against cancer cell metastasis and an early intervention measure for preventing cancer metastasis (29). However, there is a limited prognostic model based on anoikis for predicting the prognosis and personalized treatment of BC.

By focusing on the process of anoikis—programmed cell death triggered by cellular detachment—AIDAS provides novel insights into how resistance to anoikis is linked to cancer aggressiveness and metastasis. Here, we discuss the clinical implications, biological rationale, and limitations of AIDAS, and outline directions for future research that could further enhance its utility as a personalized medicine tool.

By focusing on the process of anoikis—programmed cell death triggered by cellular detachment—AIDAS provides novel insights into how resistance to anoikis is linked to cancer aggressiveness and metastasis. Here, we discuss the clinical implications, biological rationale, and limitations of AIDAS, and outline directions for future research that could further enhance its utility as a personalized medicine tool. AIDAS leverages machine learning to capture complex interactions among anoikis-related genes, enabling us to explore how gene expression patterns associated with anoikis resistance influence breast cancer prognosis. Anoikis resistance is a critical step in metastasis, and understanding its molecular underpinnings could provide pathways for intervention in cancer progression. By identifying gene clusters and pathways linked to anoikis resistance, AIDAS deepens our understanding of this biological process and its role in breast cancer outcomes, highlighting potential targets for future therapeutic strategies that could re-sensitize tumor cells to anoikis. This mechanistic insight underscores the value of combining molecular biology with advanced computational techniques to address complex questions in cancer biology.

Immunotherapy is found to be more beneficial for low-AIDAS patients by studying the immune cell infiltration score and immune checkpoint count of patients in two AIDAS subgroups. To effectively examine which patient would be more sensitive to immunotherapy, multiple analyses were utilized, and it was concluded that low-AIDAS populations have greater advantages in the treatment of ICIs, especially in response to PD-1, PD-L1, and CTLA4 drugs. For aggressive subtypes like triple-negative breast cancer (TNBC), which frequently exhibit poor responses to chemotherapy, AIDAS could be a valuable tool for tailoring immunotherapy. By stratifying TNBC patients based on AIDAS and PD-L1 expression, clinicians may be able to identify those more likely to benefit from PD-1-targeted therapies, potentially improving outcomes in this difficult-to-treat population. Enhancing the patient’s immune response to tumors by blocking the inhibitory signals of the human anti-tumor response is recognized as the most promising new cancer immunotherapy currently. CTLA-4 and PD-1 are considered two important checkpoints of the immune system, playing a negative regulatory role in the immune response of T cells. *In vivo* mouse experiments indicate that CTLA-4-dependent antibodies bind to Fc receptors rather than blocking the action of CTLA-4/B7, demonstrating the anti-tumor effect of CTLA-4 antibodies (30). Nikhil Joshi stated that PD-1 plays a crucial role in preventing T cells from attacking normal tissues in healthy individuals, and this finding may help look for a way to reduce or prevent the side effects of immunotherapy (31). Our study observed that patients in the low-AIDAS group tend to have lower PD-L1 expression, correlating with a less immunosuppressive tumor microenvironment. This reduced immune suppression may explain their improved responses to PD-1/PD-L1 inhibitors, as these therapies rely on reactivating the immune system to recognize and target cancer cells. Beyond PD-L1 expression levels, differences in the immune cell landscape and functional activity within the tumor microenvironment likely contribute to these divergent responses. Studies have shown that functional characteristics, such as T-cell activation and the presence of regulatory T-cells, can significantly impact immunotherapy effectiveness (32). Techniques such as leukosome isolation and single-cell profiling could further elucidate the immune cell dynamics within AIDAS groups, providing deeper insights into how these functional immune variations drive therapeutic responses.

Chemotherapy plays an important role in the treatment of tumors in the clinic. To study the chemotherapy efficacy among different patients, therapeutic targets and drugs were screened. After a series of analyses, it was found that BC patients with high-AIDAS are more suitable for chemotherapy. Finally, seven therapeutic targets and one drug were identified to improve the prognosis. These studies have demonstrated the effectiveness of methotrexate. For example, Methotrexate chemotherapy can induce the dysregulation of three types of glial cells, which forms the basis for chemotherapy-related cognitive impairment (33). Shen Y et al. reported that patients showed a good prognosis after they received four courses of methotrexate chemotherapy (34). Thomas S et al. believe that methotrexate is a promising drug for treating myeloproliferative tumors (35). Overall, the therapeutic potential of methotrexate has been repeatedly verified.

The genomic alterations identified in high-AIDAS tumors provide a biologically plausible explanation for the poorer prognosis associated with high anoikis resistance. High-AIDAS tumors frequently exhibit amplification of known oncogenes, such as MYC, and deletions in tumor suppressor genes, linking AIDAS with oncogenic pathways that drive tumor progression and therapeutic resistance. These findings add credibility to AIDAS as a prognostic tool, as they align with established mechanisms of cancer progression. Further exploration of these genetic drivers, within the context of AIDAS, could yield new insights into specific molecular targets, particularly for therapies aimed at reversing anoikis resistance.

Compared to other prognostic models, AIDAS offers a unique focus on anoikis-related gene expression patterns, which are particularly relevant in the context of metastasis and therapeutic resistance. Existing models tend to emphasize overall survival predictors or molecular subtypes without specifically addressing the role of anoikis and immune markers in treatment selection. AIDAS fills this gap by providing actionable insights that could directly influence treatment planning, such as recommending chemotherapy for high-AIDAS patients and immunotherapy for low-AIDAS patients. This targeted approach enhances the individualization of breast cancer treatment, which could improve outcomes by reducing unnecessary treatments and optimizing therapeutic choices based on tumor biology.

Despite the potential of AIDAS, several limitations need to be addressed. Firstly, the study’s retrospective and observational design restricts our findings to associations, without the ability to infer causality. Prospective studies with standardized, long-term follow-up would be essential to confirm AIDAS’s clinical relevance over time. Additionally, our IHC validation was conducted on a limited sample size of 30 tissue samples, which, although consistent with broader dataset findings, may not fully capture population-level heterogeneity. Expanding IHC validation to larger, multi-cohort studies would strengthen the generalizability of our results.

Our study also integrated data from multiple cohorts, each with potential variations in sample processing. Although we applied normalization and batch correction, residual technical variability may influence the findings. Future studies with harmonized, single-cohort data could provide a more uniform validation. Finally, while our bioinformatics analysis identified potential therapeutic targets through in silico drug screening, wet lab validation is essential to confirm these findings. Future research should incorporate *in vitro* and *in vivo* experiments to validate AIDAS-predicted drug responses and explore the efficacy of novel anoikis-targeting therapies.

Furthermore, the integration of AIDAS with PD-L1 expression and other immune markers offers a promising approach for precision oncology. For instance, stratifying TNBC patients by AIDAS and PD-L1 levels could help personalize immunotherapy choices, optimizing patient selection for anti-PD-1/L1 treatments. By combining molecular and immune landscape data, AIDAS represents a step towards fully personalized breast cancer management, offering a comprehensive molecular profile to guide treatment.

AIDAS exemplifies the potential of combining mechanistic understanding with machine learning to advance personalized medicine. By linking anoikis resistance with breast cancer prognosis and therapy response, AIDAS provides an actionable framework for individualized treatment selection in clinical settings. Future studies integrating multi-omics data, single-cell immune profiling, and *in vivo* validation will be crucial to refine AIDAS and maximize its clinical impact. These steps could ultimately lead to new therapeutic avenues, including anoikis-targeting agents and immunotherapy combinations, further expanding the clinical utility of AIDAS in breast cancer care.

## Conclusion

In conclusion, this study advocates for a more nuanced understanding of the TME, suggesting that the interrelationships and functional states of different immune components can significantly influence the efficacy of immunotherapy. It underscores the potential of integrating comprehensive immune profiling into clinical decision-making to tailor immunotherapeutic strategies more precisely. The differential response to immunotherapy in breast cancer groups highlights the importance of considering qualitative and functional aspects of immune cells, beyond their numerical abundance. This approach could lead to more personalized and effective therapeutic interventions, particularly in the realm of immunotherapy.

## Data Availability

The datasets presented in this study can be found in online repositories. The names of the repository/repositories and accession number(s) can be found in the article/**Supplementary Material**.
