# Supplementary material for: Machine learning based anoikis signature predicts personalized treatment strategy of breast cancer
Source: Front Immunol. 2024 Nov 22;15:1491508. doi: 10.3389/fimmu.2024.1491508 (PMC11621045; doi:10.3389/fimmu.2024.1491508)
Supplement: Supplementary file 1 [file DataSheet1.pdf]

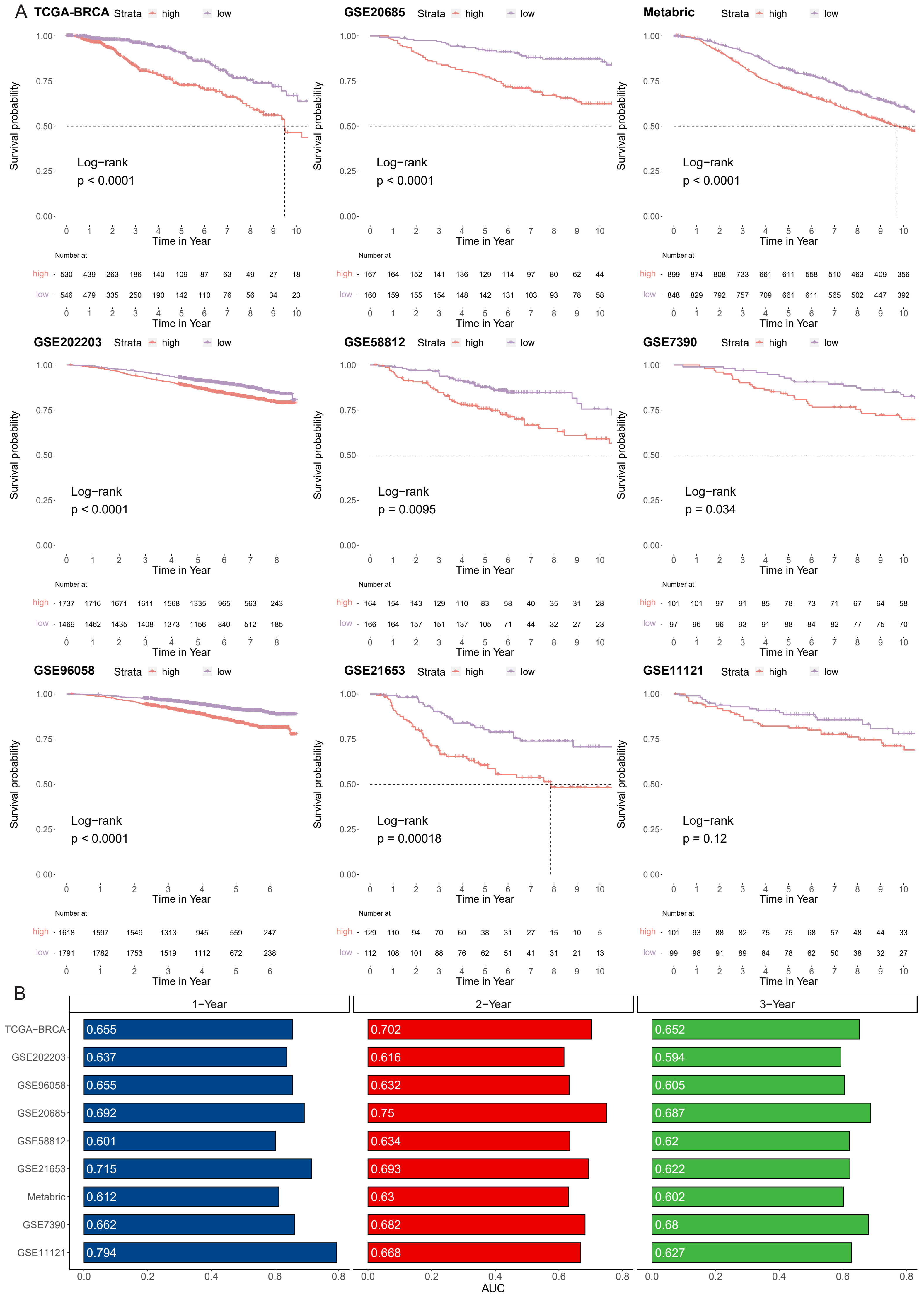

Figure S1. Evaluation of AIDAS in 9 cohorts. (A) Kaplan-Meier curves of the AIDAS in 9 cohorts. (B) Time-dependent ROC analysis for predicting OS at 1, 3, and 5 years.
